# Biomimetic Artificial Membrane Permeability Assay over Franz Cell Apparatus Using BCS Model Drugs

**DOI:** 10.3390/pharmaceutics12100988

**Published:** 2020-10-19

**Authors:** Leonardo de Souza Teixeira, Tatiana Vila Chagas, Antonio Alonso, Isabel Gonzalez-Alvarez, Marival Bermejo, James Polli, Kênnia Rocha Rezende

**Affiliations:** 1 Institute of Pharmaceutical Sciences, Goiânia, Goiás 74175-100, Brazil; leonardo.teixeira@icf.com.br; 2 Laboratory of Biopharmacy and Pharmacokinetics (BioPK), Faculty of Pharmacy, Federal University of Goiás, Goiânia, Goiás 74175-100, Brazil; tatianavchagas@gmail.com; 3 Institute of Physics, Federal University of Goiás, Goiânia, Goiás 74175-100, Brazil; alonso2233@gmail.com; 4 Department of Engineering, Pharmacy Section, Miguel Hernandez University, 03550 Alicante, Spain; isabel.gonzalez@goumh.umh.es (I.G.-A.); mbermejo@umh.es (M.B.); 5 Faculty of Pharmacy, University of Maryland, Baltimore, MD 21021, USA; jpolli@rx.umaryland.edu

**Keywords:** Franz–PAMPA, BCS drugs, biomimetic membrane, Franz cell, passive drug transport

## Abstract

A major parameter controlling the extent and rate of oral drug absorption is permeability through the lipid bilayer of intestinal epithelial cells. Here, a biomimetic artificial membrane permeability assay (Franz–PAMPA Pampa) was validated using a Franz cells apparatus. Both high and low permeability drugs (metoprolol and mannitol, respectively) were used as external standards. Biomimetic properties of Franz–PAMPA were also characterized by electron paramagnetic resonance spectroscopy (EPR). Moreover, the permeation profile for eight Biopharmaceutic Classification System (BCS) model drugs cited in the FDA guidance and another six drugs (acyclovir, cimetidine, diclofenac, ibuprofen, piroxicam, and trimethoprim) were measured across Franz–PAMPA. Apparent permeability (Papp) Franz–PAMPA values were correlated with fraction of dose absorbed in humans (Fa%) from the literature. Papp in Caco-2 cells and Corti artificial membrane were likewise compared to Fa% to assess Franz–PAMPA performance. Mannitol and metoprolol Papp values across Franz–PAMPA were lower (3.20 × 10^−7^ and 1.61 × 10^−5^ cm/s, respectively) than those obtained across non-impregnated membrane (2.27 × 10^−5^ and 2.55 × 10^−5^ cm/s, respectively), confirming lipidic barrier resistivity. Performance of the Franz cell permeation apparatus using an artificial membrane showed acceptable log-linear correlation (R^2^ = 0.664) with Fa%, as seen for Papp in Caco-2 cells (R^2^ = 0.805). Data support the validation of the Franz–PAMPA method for use during the drug discovery process.

## 1. Introduction

Favorable absorption, distribution, metabolism, and excretion (ADME) of orally administrated drugs are essential for therapeutic activity in vivo. Poor oral bioavailability contributes to a very high failure rate during pre-clinical drug development [1,2]. In this regard, the Biopharmaceutic Classification System (BCS) proposed by Amidon and co-workers [3] have been widely used as an important tool to support early drug development [4,5,6]. For orally administered drugs, gastrointestinal physiology is a key factor impacting on the rate and extent of drug absorption [7]. Transcellular passive diffusion across membranes is the major route and is governed by several molecular properties such as partition and distribution coefficient, as well as molecular weight [8,9].

Currently, important tools based on physicochemical properties and in vitro assays are used to predict in vivo gastrointestinal absorption [10]. In vitro methodologies include animal [11,12] or human tissues [13], cultured cells [14,15] and artificial membranes [16,17,18]. The Caco-2 cell monolayers in vitro model is thoroughly studied and generally mimics major transport pathways in the gastrointestinal tract [19]. However, this method is limited by long cell growth and differentiation cycles, risks of microbial contamination, and high implementation costs [19,20,21].

Cell-free permeation systems using artificial membranes are gaining progressively more interest as an alternative model to cell-based systems that can be simpler, less time consuming, and cost-effective [22,23]. Depending on the composition of the barrier, it can be classified as biomimetic barrier which is constructed from (phospho)lipids or, alternatively, from non-biomimetic barrier containing dialysis membrane [24].

Particularly, there is a growing interest in PAMPA studies with direct comparisons to Caco-2 cells using a consistent number of drugs displaying equally well prediction of in vivo data between them [25]. In this regard, major differences of key components amid cell-free membranes currently used in permeability systems was highlighted by Berben et al. (2018) [23].

**Figure 1:**
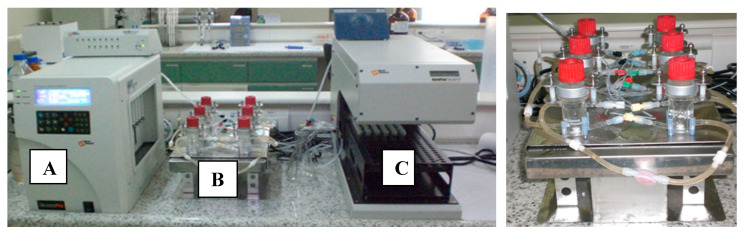
Franz cells apparatus (Microette™, Hanson Research) mounted with a previously validated PAMPA membrane from Corti et al. [5,16] for simulating gastrointestinal permeation. (**A**) System control for injection pistons of upper chambers; (**B**) upper and lower diffusion chambers with temperature and stirring control; (**C**) automated module for sampling and collection.

Here, a previously validated biomimetic artificial permeability membrane comprising of a microfilter impregnated by a phospholipid solution [5] was mounted on horizontal Franz-cells diffusion chambers (Microette™, Hanson Research) [20]. This new setup approach, herein called Franz–PAMPA (Figure 1), was challenged to assess permeability of BCS model drugs simulating gastrointestinal permeation. Therefore, the aim of this study was to validate this Franz–PAMPA system by evaluating the correlation power between apparent permeability (Papp) for BCS model drugs to their fraction of drug absorbed (Fa%) in humans for rapid and reliable information about passively transported drugs [25,26].

## 2. Materials and Methods

### 2.1. Materials

Membrane supports were purchased from Millipore^®^ (Mixed Cellulose Esters VCWP 047000; 0.1 µm × 47 mm, white plain, New York, NY, USA). All 19 compounds for permeation studies (acyclovir, amoxicillin, atenolol, caffeine, cimetidine, diclofenac, furosemide, hydrochlorothiazide, ibuprofen, mannitol, metoprolol, naproxen, piroxicam, propranolol, ranitidine hydrochloride, trimethoprim, and verapamil hydrochloride) were of analytical grade and kindly supplied by ICF (Pharmaceutical Sciences Institute, Goiânia, Brazil). All organic solvents were of HPLC grade and solid reagents were of analytical grade.

The spin labels 5-doxyl stearic acid (5-DSA) and 16-doxyl stearic acid (16-DSA) used for electron paramagnetic resonance (EPR) spectroscopy were purchased from Sigma-Aldrich Chem Co. (St. Louis, MO, USA). The spin labels 1-palmitoyl-2-stearoyl-(5-doxyl)-sn-glycero-3-phosphocholine (5-PC) and 1,2-dipalmitoleoyl-sn-glycero-3-phosphocholin (16-PC) were purchased from Avanti (Avanti Polar Lipids, Inc., Alabaster, AL, USA).

### 2.2. Methods

#### 2.2.1. Impregnation of Membrane Support

Membranes were impregnated by immersion for 60 min (22 ± 1 °C) with a lipid solution (mixture of phospholipids), as previously reported [5]. Briefly, the lipid phase solution for impregnation was a mixture of 1.7% phospholipids (Lipoid^®^ E 80, Ludwigshafen, Germany), 2.1% cholesterol (Sigma–Aldrich Chemical Co., Milan, Italy), and 96.2% n-octanol (Synth, Diadema, Brazil). Excess lipid was absorbed with cellulose filter paper over 30 min. Next, all impregnated membranes (N = 20) were weighed on a microanalytical scale (Mettler Toledo, mod. XPE56DR, Columbus, OH, USA) and evaluated to check for its accuracy (211.2 mg ± 6.0%). Prior to use, impregnated membranes were protected from moisture atmosphere and refrigerated (−8 °C, 24 h). It is worth mentioning that all membranes were stabilized prior to use. Stabilization was confirmed by EPR spectra which did not show any signals of physicochemical degradation: none of membranes showed any difference on ^14^N-hyperfine coupling constant value (14.8 G) demonstrating its stability [25]. EPR signals were compared just after 24 h of refrigeration and post-run permeability studies as well as after a month of refrigerated storage time (*data not shown*).

#### 2.2.2. Electronic Paramagnetic Resonance (EPR)

The biomimetic membranes were impregnated, as described above. Spin labeling technique was employed to examine the conformational structure of the membrane using 5-DSA or 16-DSA. EPR was performed using a Bruker ESP 300 spectrometer (Bruker, Rheinstetten, Germany) equipped with an ER 4102 ST resonator. The instrument settings were microwave power of 2 mW; modulation frequency of 100 KHz; modulation amplitude of 1.0 G; magnetic field scan of 100 G; sweep time of 168 s; and a detector time constant of 41 ms. EPR spectral simulations were performed using the nonlinear-least-squares (NLLS) program for an isotropic model. The biomimetic membrane was introduced into flat, quartz EPR cell to perform the EPR measurements at room temperature (~25°C).

#### 2.2.3. Permeation Studies

Permeation studies were performed using a Franz vertical diffusion cell (MicroettePlus, Hanson Research, CA, USA). Impregnated artificial membranes (Franz–PAMPA) were positioned between upper and lower part of diffusion cells and, the donor (1 mL) and receptor (7 mL) compartments holding phosphate-buffered solution (PBS) pH 7.4 (USP 32). In order to minimize the unstirred water layer (UWL), receptor compartment media was stirred (500 rpm). The temperature was kept constant (37.0 ± 0.5 °C). Each drug (n = 3) was added in the donor compartment at a fixed concentration (=10 mg/mL). One milliliter of saturated drug solutions was transferred to the donor compartments and capped to prevent evaporation. The experiments were performed under ‘infinite dose’ conditions [26,27], except for caffein, metoprolol, propranolol, naproxen, ranitidine, and atenolol (D_0_ ≤ 0.01). Individual drug solubility is further shown in results section. Metoprolol was used as a low/high BCS permeability class boundary reference drug for the Franz–PAMPA assay [28].

Samples from permeation studies were collected during 12 h (0.25; 0.5; 1.0; 2.0; 3.0; 4.0; 5.0; 6.0; 10.0, and 12.0 h) and analyzed by HPLC (Shimadzu Class VP; Kyoto, Japan or Agilent 1220, Santa Clara, CA, USA) according to official compendiums (USP 32 or Brazilian Pharmacopeia 4th edition). The sampling volume was immediately replaced with the same volume of fresh PBS prewarmed solution at 37° ± 0.5 °C. Calibration curves were performed at least at three concentration levels for each drug tested, in a GLP-accredited laboratory (Institute of Pharmaceutical Sciences, Goiânia, Goiás, Brazil). The validated chromatographic conditions used for the drug permeability assay are given in Table 1.

#### 2.2.4. Permeability Calculations

**Figure 2:**
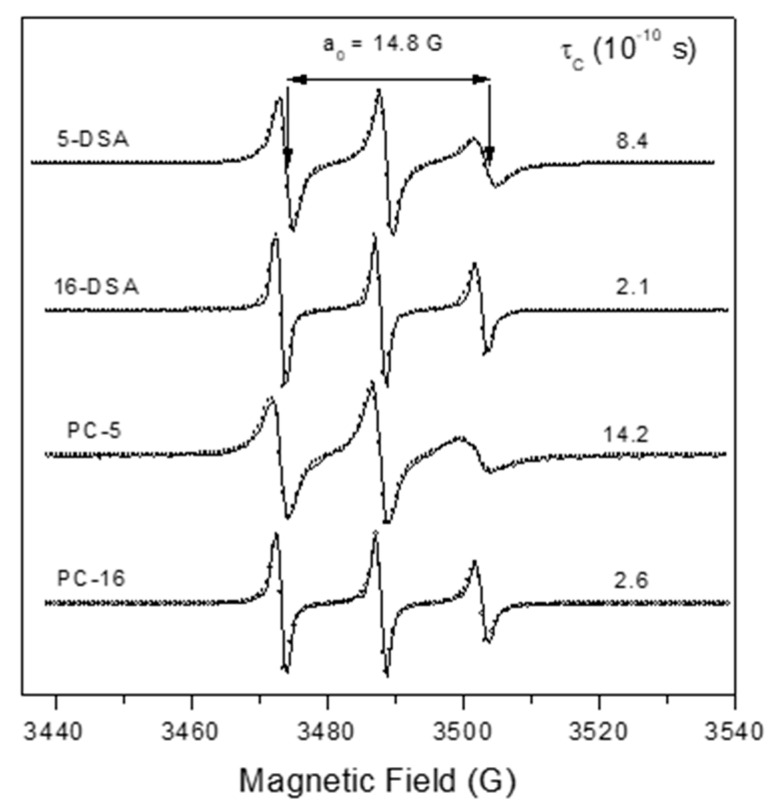
Experimental (solid line) and best-fit (empty circles) EPR spectra for several spin labels in BAMPA. The isotropic 14N-hyperfine coupling constant, a_0_, showed equal spectra values of 14.8 G, consistent with a spin label in phospholipidic bilayer of eukaryotic cells. The rotational correlation time value, τC, is also showed. 5-DSA or 16-DSA: 5- or 16-doxylstearic acid); 5-PC or 16-PC:5-0r 16-phosphatidylcholine).

The diffusion area (*A*) was calculated from the radius of the Franz cell and was 1.77 cm^2^. Flux through membrane to receptor compartment (*J*; µg/cm^2^/s) was calculated by dividing the amount of drug accumulated in the receptor compartment by *A*. The Fick’s first law was derived to calculate flux (*J*) at steady state (Equation (1)):

*J* = dQ/dt*A (1)

where *dQ* is the amount of drug across the membrane (in moles), *dt* the permeation time (in seconds), and *A* the diffusion area (in cm^2^). Note that *J* was obtained from the slope of the curve at steady state from typical mean cumulative concentration-time plots (minimum of triplicates), as further shown in results section (Figure 2). Coefficient of variation (CV) of flux for each drug was also measured.

The apparent permeability (*Papp*) was calculated normalizing the flux (*J*) over the drug concentration in the donor compartment *C*_0_, as described by the following Equation (2):

*Papp*= *J/C*_0_ (2)

This approximation was used in all cases, even when sink conditions do not hold and donor concentrations change with time, as already described for some experiments [29]. In addition, the following equation was used to account for the fact that in most cases sink conditions were not maintained [30].

(3) $$C_{receiver,t}=\frac{Q_{total}}{V_{receiver}+V_{donor}}+\left(\left(C_{receiver,t-1}\cdot f\right)-\frac{Q_{total}}{V_{receiver}+V_{donor}}\right)\cdot e^{-P_{eff}\cdot S\cdot \left(\frac{1}{V_{receiver}}+\frac{1}{V_{donor}}\right)\cdot \Delta t}$$

where *C_receiver_*_,*t*_ is the drug concentration in the receiver chamber at time *t*, *Q_total_* is the total amount of drug in both chambers, *V_receiver_* and *V_donor_* are the volumes of each chamber, *C_receiver,t−1_* is the drug concentration in receiver chamber at previous time, *f* is the sample replacement dilution factor, *S* is the surface area of the membrane, Δ*t* is the time interval and *P_eff_* is the permeability coefficient. This equation considers a continuous change of the donor and receiver concentrations, and it is valid in either sink or non-sink conditions. The curve-fitting is performed by non-linear regression, by minimization of the sum of squared residuals (*SSR*), where:

(4) $$SSR=\sum^{\;}{\left[C_{r,i,obs}\;-\;C_{r,i}\left(t_{end,i}\right)\right]}^{2}$$

*C_r,i,obs_* is the observed receiver concentration at the end of interval *i*, and *C_r,I_(t_end,i_)* is the corresponding concentration at the same time calculated according to Equation (3) [29].

Classification as high permeability was established if the calculated permeability (under sink or non-sink conditions was higher than 0.8* metoprolol permeability [31].

The in vitro permeability (Papp) of each drug studied was compared to in vivo absorption in humans (Fa%), Papp in Corti artificial membrane [16], and Papp in Caco-2 cells.

## 3. Results

### 3.1. EPR Analysis and Membrane Stability

The Franz–PAMPA was characterized by EPR spectroscopy of lipid spin labels of doxyl class. The spectra showed a movement consistent with lipid bilayer (Figure 1). Two analogs of stearic acid, 5-DSA and 16-DSA, and two analogs of phosphatidylcholine, 5-PC and 16-PC, having the nitroxide radical positioned at the 5th and 16th carbon atom of the acyl chain, respectively, were used to examine the molecular dynamic at two regions into the bilayer. The EPR spectra of these four spin labels are shown in Figure 2.

The EPR parameter—isotropic ^14^N-hyperfine coupling constant, a_0_—increased with increasing dielectric constant (i.e., solvent polarity) in which the nitroxide radical is dissolved. The measured value of 14.8 G is consistent with a spin label in a membrane [32]. The spin labels 5-DSA and 5-PC with the nitroxide moiety in the region near the polar head group of the bilayer showed more restricted rotational motion relative to their positional isomers 16-DSA and 16-PC, in which the nitroxide radical is more deeply inserted in the hydrophobic core. These results indicate the existence of a gradient of flexibility along the acyl chain, with more restricted motion in the polar region. This pattern is consistent with the properties of lipid bilayers from eukaryotic cells. The rotational motion at the polar interface of the membrane was more restricted for the spin label analog of phosphatidylcholine (5-PC) with τ_C_ of 14.2 × 10^−10^ s than for the stearic acid one (5-DSA) whose τ_C_ was of 8.4 × 10^−10^ s (Figure 2).

Membrane barriers from similar models such as PAMPA and PVPA have been proven to be stable in a pH range from 2 to 8 [33]. Here, EPR spectra were also recorded before and after permeation studies to check for the integrity of biomimetic membranes. No leaching of barrier-constituents such as phosphatidylcholine and lipids into the donor compartment could be evidenced as none of membranes showed any difference on ^14^N-hyperfine coupling constant value (14.8 G) demonstrating its stability [34]. Likewise, using the same chemical composition as Corti (2006) [5], acidic and basic drugs also showed pH-dependent permeability according to the pH partition theory [25,35]. Accordingly, close Person’s correlation coefficient was seen (r = 0.7355) to our data from Franz–PAMPA *versus* PAMPA pH 7.4 data from literature.

In this regard, pHs of drug solutions were all measured to assure buffer capacity and drug stability. Some authors correlated membrane flux with the fraction absorbed in human, showing that the flux through the egg lecithin/dodecane membrane correlated better than octanol/water logD values with the fraction absorbed in humans [17]. Later, an in-depth investigation of pH impact on drug Franz–PAMPA permeability will be necessary to increase the biomimetic and absorption predictive power of this method, although the study of this factor was beyond the scope of this work.

### 3.2. Membrane Validation and Performance

Studies here deals with a modified PAMPA method over Franz cell apparatus. The biomimetic membrane (Franz–PAMPA) has been previously described by Corti and coworkers [5] as a modified version from Kansy et al. (1998) [36]. Mannitol and metoprolol were used as a marker for the cutoff point between low and high permeability drugs.

The apparent permeability coefficient (Papp) values found for mannitol and metoprolol, over the lipid impregnated membrane (2.27 × 10^−5^ and 2.55 × 10^−5^ cm/s, respectively) were higher when compared to the non-impregnated one across Franz–PAMPA (3.20 × 10^−7^ and 1.61 × 10^−5^ cm/s, respectively), indicating the resistivity of the lipid membrane itself.

Membrane performance was assessed using 14 representative model drugs (Table 2) cited in the FDA BCS guidance [37]. Class I model compounds were caffeine, metoprolol, propranolol and verapamil. Class II model compounds were diclofenac, ibuprofen, naproxen and, piroxicam. Class III model compounds were atenolol, cimetidine, ranitidine, and trimethoprim. Class IV model compounds were acyclovir, furosemide and hydrochlorothiazide. This classification was based in the permeability class indicated in the FDA guidance [37] and, on solubility from literature [28,38].

**Figure 3:**
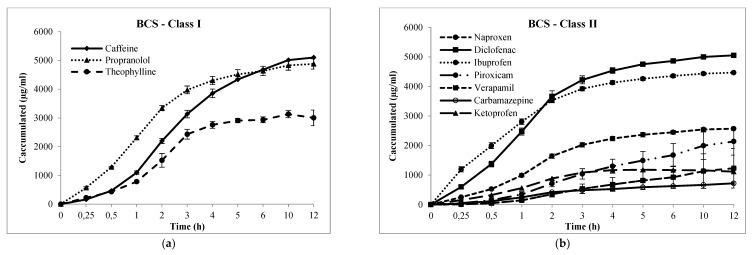

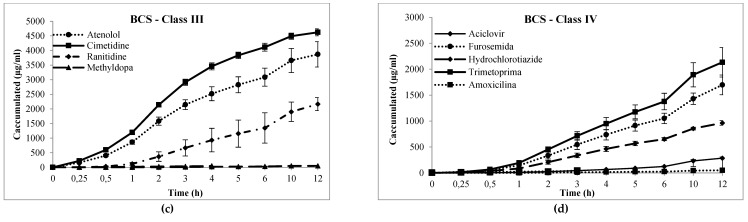
Cumulative transport (µg/mL, h) of drugs across Franz-PAMPA: (**a**) BCS class I; (**b**) BCS class II; (**c**) BCS class III; and (**d**) BCS class IV. Permeability was calculated from the linear portion (R^2^). Data are presented as mean ± SD, n = 3

Cumulative drug transport through Franz–PAMPA was plotted over 12 h and the Papp was calculated from the slopes obtained from linear regressions (Figure 3, Table 2). Of the 21 compounds studied by Corti and coworkers and of the 14 compounds studied here, there were 11 common compounds tested in both studies: acyclovir, atenolol, caffeine, cimetidine, furosemide, hydrochlorothiazide, metoprolol tartrate, naproxen, propranolol, ranitidine, and trimethoprim. For these drugs Caco-2 Papp values were also surveyed from literature and compared here (Table 2).

For high permeability drugs (BCS I and II, Table 2), the Papp values showed to be in the range of 4.6–75.2 × 10^−6^ cm/s in Franz–PAMPA. For Caco-2 assay, values were narrower (15.8–52.5 × 10^−6^ cm/s) and, for Corti membranes they were most narrow (39.7–48.8 × 10^−6^ cm/s).

**Figure 4:**
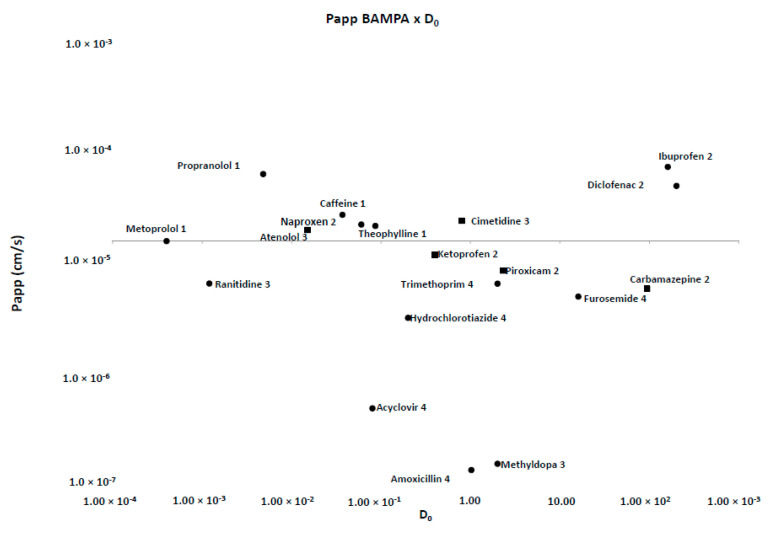
Permeability values for Franz-PAMPA *versus* D_0_ drugs using metoprolol as reference drug. Most drugs (●; 14 out of 19) were in accordance with previous Biopharmaceutic Classification System (BCS) classification. Some of them (■; 5 out of 19) disagreed.

For low permeability drugs (BCS III and IV, Table 2), the Papp coefficient found were consistently much lower than high permeability drugs. Franz–PAMPA, Caco-2 [20,39], and Corti membrane provided value ranges of 0.2–24.6 × 10^−6^ cm/s, 0.1–83.0 × 10^−6^ cm/s, and 3.2–45.5 × 10^−6^ cm/s, respectively. Permeability of most drugs tested here showed Papp >1.0 × 10^−5^ cm/s (Figure 4).

Typically, PAMPA methods are affected by high variability, and therefore, data can be somehow noisy for poorly permeable drugs. Variability is also an issue that impacts permeability for Caco-2 [5] and other in situ [19] and in vivo [24] models. For low permeability drugs (Fa < 80%), Avdeef and coworkers (2003) [6] measured variability for more than 200 different drugs accounting for more than 600 measurements. Papp values close to 10 × 10^−6^ cm/s showed variability of around 10%. Such error can increase slightly for higher Papp values but is larger for Papp < 0.1 × 10^−6^ (60%), with 0.01 × 10^−6^ values exhibiting variability of 100% or more. Although, currently BBB (blood brain barrier) [40] or Skin-PAMPA [41] methods can achieve higher precision and reproducibility with some other controlled protocols. Specific adjustments include setting incubation time as low as possible, increasing sensitivity of analytical methods, controlling membrane homogeneity either on the filter or among filters, besides the rationale for compounds dataset amongst others.

Likewise, permeability of small hydrophilic compounds is frequently underestimated in PAMPA since the membrane has hydrophobic nature besides being a cell-free system [42]. For the FDA-listed drugs, PAMPA Papp displayed values ranged from 0.00 to 2.35 × 10^−5^ cms^−1^, indicating it was not sensitive enough to discriminate and rank poorly permeable compounds. In contrast, Franz–PAMPA showed values in a wider Papp range of 0.4–68.1 × 10^−6^ cm/s. This could be tentatively explained due to the hydrophilic nature of membrane support and pH-dependent characteristics of the drugs [22,24,31]. Moreover, Franz cell stirring clearly reduces the unstirred water layer resistance in the system.

**Figure 5:**
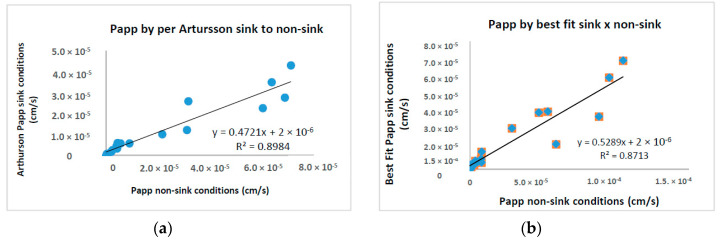
Papp calculations using equations by (**a**) per Artursson *non-sink versus sink* conditions and, (**b**) per Artursson *non-sink* compared to *sink* conditions best fit method of the linear portion.

Additionally, variability of Papp values was also addressed by the calculation methods. A more sophisticated analysis is done using Artursson’s equation [15] for sink and non-sink conditions as well as checking the impact of extracting a permeability coefficient from data that are not at true steady state and, thus, possibly impacted by dose depletion. Note that for both the sink and the non-sink equation, Papp values showed a particularly good correlation between them (0.8984). Similarly, Papp values obtained by us showed to be very alike to values calculated according to Artursson’s non-sink equation (Table 2, Figure 5). The reason is that we used the same systematic procedure, i.e., the best fit method through the linear portion, to calculate all the slopes characterizing an accurate permeability flow, so that the impact from dose depletion is considered not above average. As a result, all drugs got the same BCS classification in both methods.

**Figure 6:**
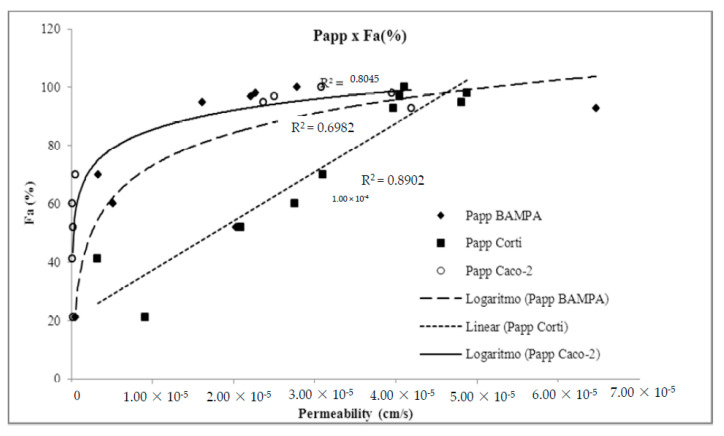
Demonstration of method suitability from Franz–PAMPA assay permeability and fraction of dose absorbed in humans (Fa%) compared to Caco-2 cells (ο) and Corti membrane (■). Actively transported drugs were removed for R^2^ calculation. Corti membrane Papp (◆) correlation to %Fa (R^2^ = 0.890) was essentially unchanged.

In this context, Franz–PAMPA profile is mimicking biological permeation in a graphical pattern related to permeation through Caco-2 cells (R^2^ = 0.826). Obtained Papp values *versus* fraction of dose absorbed in humans (Fa%) showed log linear correlation (Figure 6), as also described by Zhu et al. [38] when analyzing permeability performance of 93 commercial drugs as for artificial membranes. As expected, Franz–PAMPA also showed a significantly improved log linear correlation (R^2^ = 0.6982) when actively transported compounds ranitidine, trimethoprim, and verapamil were not incorporated in the regression analysis. In contrast, the Fa% *versus*. Corti membrane correlation was linear (R^2^ = 0.904). Such discrepancy from Franz–PAMPA and Caco-2 reveals that passive permeability of tested drugs through Corti membrane was greater and better suitable, especially for low and moderate permeability drugs, as discussed elsewhere [39]. PAMPA and Caco-2 technique would be best suited for compounds with medium and high permeabilities. For low permeability compounds, small differences in measured Papp are expected to yield large differences in Fa% values resulting in imprecise measurements.

Currently, a promising biomimetic barrier also adapted to Franz diffusion cells Permeapad™— [22] was reported for six drugs concurrent to our model (acyclovir, atenolol, caffeine, ibuprofen, and metoprolol). Even if a satisfactorily comparative analysis was not straightforward, BCS classification of most drugs (4 out of 5) showed to be identical with similar Papp rank order (Table 2).

## 4. Conclusions

The Franz–PAMPA method provided a permeability pattern similar to those from Caco-2. Methodologically, the advantages of Franz–PAMPA over Caco-2 are the lower costs and simplicity of membrane preparation (e.g., reagents and artificial membrane are commercially available). Furthermore, the method is very versatile and could be transformed in a high-throughput in vitro method to detect and classify compounds absorbed by passive diffusion.

Using metoprolol as a high permeability marker (Papp = 1.61 × 10^−5^ cm/s; Figure 2), seven drugs were classified as highly permeable (best fit method): atenolol, caffeine, cimetidine, diclofenac, ibuprofen, naproxen, and propranolol (Table 2). Only atenolol and cimetidine were misclassified as highly permeable drugs, relative to their prior literature classification as BCS 3 drugs.

Additionally, 10 out of 17 drugs were classified as low permeability drugs in Franz–PAMPA. Nevertheless, only naproxen, piroxicam, and verapamil (3 out of 10) had their permeability underestimated according to BCS, as they performed as low permeability drugs instead of BCS2 drugs.

Summing up, a potential limitation of our study is that the Papp values were calculated with an equation in which the underlying assumptions are constant donor concentration and sink conditions. In order to account for that, we also did the calculations to estimate permeability values under non-sink conditions. The obtained values are about the same compared with the true values (i.e., assuming donor concentration change and non-sink conditions). Although the relative estimation error does change across high *versus* low permeability compounds [29], the practical implications for predicting oral fraction absorbed would only be a “shift” to the left on the abscissa. In the case of a direct correlation with Caco-2 values, it would be reflected in a different slope, but it would not change the significance of the regression line. In the case of the use of the apparent permeabilities for classification of compounds, the reference value of metoprolol is also underestimated, so the classification outcome would not be changed [29].

As a final comment, the ability of Franz–PAMPA to classify drugs was good and can be potentially challenged at different pH conditions to predict intestinal permeability of drugs showing passive transport. Eventually, the Franz–PAMPA cell diffusion can be modulated in lipid composition and may be a suitable alternative for studying other biological barriers such as blood–brain barrier, skin, and mucosal barriers as buccal or nasal. The current dataset adds valuable information for future analysis of drug-molecular interactions at the lipid layer and in silico model development. Additionally, all apparatus and supplies experimentally used on Franz–PAMPA are commercially available and affordable to facilitate drug discovery method application.

## Figures and Tables

**Table 1:** Pharmacopeial methods applied on drug analysis and their respective limit of quantification (LOQs).

Drug	Chromatographic Conditions (Stationary and Mobile Phase; λ; Flow Rate; Injection Volume)	LOQ (μg/mL)
Acyclovir	C-18 (5 µm; 250 × 4.2 mm), acetic acid: water (1:1000); 254 nm; 3.0 mL/min; 20 µL	46.3
Amoxicillin	C18 (5 µm; 250 × 4.0 mm); acetonitrile e phosphate buffer pH 5.0 (4:96); 230 nm, 1.5 mL/min, 10 μL	1.00
Atenolol	C-18 (5 µm; 300 × 3.9 mm); Dissolve 1.1 g of sodium heptane sulfonate and 0.71 g of sodium phosphate dibasic anhydrous in 700 mL of water. Add 2 mL of dibutylamine. Adjust pH 3.0. Add methanol (300 mL); 226 nm; 0.6 mL/min; 10 µL	3.4
Caffeine	C-18 (5 µm; 150 × 4.6 mm); Solution of 1.64 g anhydrous sodium acetate in 2000 mL of water. Take 1910 mL of this solution add acetonitrile (50 mL), tetrahydrofuran (40 mL). Adjust pH 4.5 with glacial acetic acid; 275 nm; 1.0 mL/min; 10 µL	19.0
Carbamazepine	CN ((250 mm × 4,.6 mm); Water, methanol, and tetrahydrofuran (85:12:3), 0.22 mL formic acid and 0.5 mL triethylamine; 230 nm, 1.5 mL/min, 20 μL	0.03
Cimetidine	C-18 (5 µm; 300 × 3.9 mm); 20% methanol in 0.3% phosphoric acid solution; 220 nm; 2.0 mL/min; 50 µL	1.0
Diclofenac sodium	C-8 (5 µm; 250 × 4.6 mm); phosphate buffer pH 2.5 and methanol (30:70); 254 nm; 1.0 mL/min; 10 µL	0.20
Furosemide	C-18 (5 µm; 250 × 4.6 mm); Water, tetrahydrofuran, and glacial acetic acid (70:30:1); 254 nm; 1.0 mL/min; 20 µL	16.6
Hydrochlor-thiazide	C-18 (5 µm; 150 × 4.6 mm); Solution A: acetonitrile and methanol (3:1). Solution B: 0.5% formic acid. Gradient: 0–3 min. Sol A: Sol B (3:97), 5–14 min. Sol A: Sol. B (3 to 36:97 to 64), 14–18 min. The Sol. A: Sol B (36 to 3:64 to 97), 18–20 min. Sol A: Sol B (3:97); 275 nm; 1.0 mL/min; 10 µL	7.8
Ibuprofen	C-18 (5 µm; 250 × 4.6 mm); 4% chloroacetic acid pH 3.0 and acetonitrile (40:60); 254 nm; 2.0 mL/min; 10 µL	13.9
Ketoprophen	C18 (3 µm; 150 × 4.6 mm); water, acetonitrile, and phosphate buffer pH3.5 (55:43:22); 233 nm, 1.0 mL/min, 20 μL	1.56
Metoprolol	C-18 (5 µm; 300 × 3.9 mm); 961 mg of pentane sulfonate, 82 mg of anhydrous sodium acetate, 550 mL of methanol, 470 mL of water and 0.57 mL of acetic acid; 254 nm; 1.0 mL/min; 30 µL	13.8
Methyldopa	C18 (5 µm; 300 × 3.9 mm); Monobasic phosphate buffer pH 3.5; 280 nm, 1.0 mL/min, 50 μL	0.12
Naproxen	C-18 (5 µm; 150 × 4.6 mm); Acetonitrile, water, and glacial acetic acid (50:49:1); 254 nm; 1.2 mL/min; 20 µL	3.6
Piroxicam	C-18 (5 µm; 250 × 4.6 mm); Buffer solution containing 7.72 g of anhydrous citric acid in 400 mL of water and 5.35 g dibasic sodium phosphate in 100 mL of water, mix the two solutions and adjust volume to 1000 mL with water.Mix buffer and methanol (55:45); 254 nm; 1.2 mL/min; 20 µL	4.0
Propranolol	C-8 (5 µm; 250 × 4.6 mm); Dissolve 0.5 g of sodium dodecyl sulfate in 18 mL of 0.15 M phosphoric acid. Add 90 mL of acetonitrile, 90 mL of methanol, dilute with water to complete 250 mL; 290 nm; 1.5 mL/min; 20 µL	8.2
Ranitidine	C-18 (3.5 μm; 10 × 4.6 mm); buffer phosphate pH 7.1: acetonitrile (80:20); 230 nm, 1.5 mL/min, 35°C, 10 μL	7.4
Trimethoprim	C-18 (5 μm; 250 × 4.2 mm); 1% glacial acetic acid: acetonitrile (21:4); 254 nm, 2 mL/min, 10 μL	0.15
Theophylline	C-18 (5 μm; 300 × 4.0 mm); 7% acetonitrile in sodium acetate buffer; 280 nm, 1 mL/min, 10 μL	0.22
Verapamil	C-18 (5 µm; 150 × 4.6 mm); 0.015 N sodium acetate in 3.3% glacial acetic acid. add acetonitrile and 2-amino-heptane (70:30:0.5); 278 nm; 0.9 mL/min; 10 µL	0.50

**Table 2:** Papp calculated values in Franz-PAMPA and using Non-Sink Artursson equation. BCS classification of studied drugs and literature data to all other parameters.

Drug	^5^ BCS	^1^ Fa(%)	Papp _×_ 10^−6^ cm/s						^5^ LogP	^5^ Log D pH 7.4	^5^ pKa	^4,5^ Intrinsic Solubility (mg/mL)
			Franz–PAMPA	Non-Sink Arthurson	^1^ Caco-2	^2^ Corti	^3^ PAMPA pH 7,4	^3^ Permeapad™				
Metoprolol	**I**	95	15.8 (HP)	59.0	23.7 (HP)	48.1 (HP)	3.5	1.0	1.9	−0.2	9.6	1000.0
Caffeine		100	36.2 (HP)	53.3	30.8 (HP)	41.1	10.8	20.4	−0.1	0.02	0.6	21.17
Propranolol		93	33.1 (HP)	88.4	41.9 (HP)	39.7	23.5	nC	2.65	1.3	9.5	33.0
Theophylline		97	22.1 (HP)	---	25.0 (HP)	40.5	--	7.2	−0.25	−0.05	0.6 & 8.55	8.33
Carbamazepine	**II**	100	5.97	---	-	-	11.3	nC	2.5	1.8	1.0 & 13.9	0.12
Diclofenac		100	68.1 (HP)	104.9	-	-	12.5	nC	4.4	1.2	3.99	0.001
Ibuprofen		93	57.5 (HP)	36.0	52.5 (HP)	-	6.8	16.6	3.1	0.7	4.9	0.01
Ketoprofen (AT)		100	12.1	---	20.1	42.7	16.7	nC	3.3	−1.51	4.5	0.051
Naproxen		98	2.89 (HP)	1.7	39.5 (HP)	48.8 (HP)	10.6	nC	3.2	0.2	4.2	33.0
Piroxicam		100	11.0	9.6	35.6 (HP)	-	8.2	nC	2.0	−0.07	2.33 & 5.1	0.11
Verapamil (AT)		98	5.39	5.0	15.8	41.6	7.4	9.3	3.8	2.7	8.9	0.44
Atenolol	**III**	52	25.8 (HP)	22.0	0.2	20.9	0.0	4.3	0.2		9.6	26.5
Cimetidine		93	35.6 (HP)	31.0	0.7	-	0.0	nC	0.4	0.4	6.8	6.0
Methyldopa		41	---	---	0.2	3.2	--	nC	0.4		1.7 & 9.9	10.0
Ranitidine (AT)		55	6.81	5.3	0.5	21.5	0.5	nC	0.3	−0.3	2.1 & 8.1	100.0
Acyclovir	**IV**	21	0.40	0.4	0.3	9.1	0.0	7.9	−1.7	−1.7	2.3 & 9.3	10.0
Amoxicillin		93	0.85	0.07	0.8	-	1.5	nC	0.9	---	3.2 & 11.7	4.0
Furosemide		60	4.57	4.5	0.1	27.5	0.6	nC	2.3	−0.7	3.5 & 10.6	0.01
Hydrochlorothiazide		70	2.74	2.7	0.5	31.0	0.1	nC	−0.1	−0.1	7.9	1.0
Trimethoprim (AT)		97	6.61	7.7	83.0 (HP)	45.5	5.0	nC	0.9	0.7	7.1	0.4

nC = non classified ^1^ Yamashita et al., 2000 [20] and Zhu et al., 2002 [39]; ^2^ Corti and co-workers, 2006 [5]; ^3^ Di Cagno et al. [22]; ^4^ Lindenberg et al. 2004 [38], ^5^ Kasim et al., 2004 [28] (AT) actively transported drugs. (HP) high permeability drug [38]—Data not available for non-sink calculations.
